# Postnatal HIV vertical transmission and the impact of infant feeding choice in the ART era

**DOI:** 10.4102/sajhivmed.v26i1.1738

**Published:** 2025-10-20

**Authors:** Victoria Ndarukwa, Mark Cotton, Moleen Zunza, Lucy Mupfumi, Hans Amukugo

**Affiliations:** 1 Division of Epidemiology and Biostatistics, Department of Global Health, Faculty of Medicine and Health Sciences, Stellenbosch University, Cape Town, South Africa; 2 Division of Paediatric Infectious Diseases, Department of Paediatrics and Child Health, Faculty of Medicine and Health Sciences, Stellenbosch University and Tygerberg Children’s Hospital, Cape Town, South Africa; 3 Botswana Harvard Health Partnership, Gaborone, Botswana; 4 Faculty of Health Sciences, School of Nursing and Public Health, University of Namibia, Oshakati, Namibia

**Keywords:** exclusive breastfeeding, breastfeeding, infant feeding, antiretroviral therapy, HIV vertical transmission

## Abstract

**Background:**

Mixed feeding (MF) among HIV-exposed infants is a common practice in sub-Saharan Africa. Evidence suggests that MF is an additional risk for postnatal HIV transmission, even with antiretroviral therapy (ART).

**Objectives:**

To determine the risk of HIV transmission by infant feeding modality.

**Method:**

We searched for studies focusing on mothers living with HIV and their infants, and their feeding modality. The primary outcome was postnatal HIV transmission.

**Results:**

Nine studies were identified from 570 reports. Overall, postnatal HIV transmission was measured at varying time points across the studies. Five studies reported transmission rates at 12 months, estimates ranged from 0% to 7%. Higher transmission rates were reported at 18–24 months, peaking at 11.6%, compared to a peak of 5% at 6 months. Adherence to maternal ART was reported in three studies ranging from 84% to 98%. Exclusive breastfeeding (EBF) duration was established in seven studies ranging from 51% to 97% at 6 months, with early complementary feeding introduced as early as 2 weeks. Two studies reported increased risk in HIV transmission associated with MF: 4–6-fold higher risk of transmission in MF compared to EBF infants.

**Conclusion:**

Reduced risk of postnatal HIV transmission was revealed in mothers on ART, and EBF, supporting WHO recommendations, two studies showed the presence of MF increased the risk of postnatal transmission. There is limited information on the actual risk of postnatal transmission associated with MF in mothers adhering to ART with suppressed viral loads.

**What this study adds:** This systematic review provides a comprehensive synthesis of quantitative literature regarding infant feeding patterns and the impact of infant feeding choices on HIV transmission in sub-Saharan Africa in the ART (Option B+) era.

## Introduction

About 44% of infants 0–6 months old are exclusively breastfed, with the majority weaning through the introduction of solid and liquid foods as early as 1 month postpartum.^1^ Mixed feeding (MF) among HIV-exposed infants without HIV is a common practice in sub-Saharan Africa.^2,3,4,5,6^ Reports suggest that MF is an additional risk for postnatal vertical HIV transmission, even with antiretroviral therapy (ART).^7,8,9^ Current World Health Organization (WHO) guidelines recommend lifelong ART for pregnant and breastfeeding women living with HIV, regardless of the CD4 count. Exclusive breastfeeding (EBF) is recommended in the first 6 months of life, with complementary feeding added until 24 months of age or beyond.^10^ The decision regarding whether mothers living with HIV should breastfeed is generally based on balancing the risk of HIV transmission through breastfeeding, with the increased risk of morbidity and death from malnutrition, diarrhoea, and pneumonia if the infants are not exclusively breastfed.

An infant born HIV negative can acquire HIV from breast milk.^7,8,9^ Prior to the introduction of ART, MF was considered a high risk for HIV vertical transmission (VT) in infants. As ART was only introduced in public health programmes in 2004, there is substantial evidence of reduced postnatal HIV transmission risk under the cover of maternal ART. Prior to 2004, WHO was concerned about HIV VT through breastfeeding, which challenged their endorsement of breastfeeding.^11^ At that time, the extent of breastmilk transmission was unknown. In 1998, the Joint United Nations Programme on HIV/AIDS (UNAIDS) recommended that the risk of HIV transmission through breastfeeding be balanced against the added risk of morbidity and mortality when infants are not breastfed.^12^ Consequently, in 2003, WHO and the United Nations Children’s Fund (UNICEF) jointly recommended an individualised approach for HIV and infant feeding, in which women living with HIV would be counselled on feeding options, according to their household circumstances.^11^ In the pre-ART era, in 1999, a South African study showed that 18.8% (95% confidence interval [CI]: 12.6–24.9) of 156 never-breastfed children acquired HIV versus 21.3% (17.2–25.5) of 393 breastfed children (*P* = 0.5). Significantly more MF than EBF infants acquired HIV by 3 months (24.1% [19.0–29.2] vs. 14.6% [7.7–21.4; *P* = 0.03]).^13^ However, for a mother on ART for the duration of breastfeeding or for life, the risk of VT reduced to 11% or less, with EBF for the first 6 months associated with a 3- to 5-fold lower risk of HIV transmission than MF.^14,15,16^ In updated guidance in 2021, the WHO and UNICEF recommended that in settings where morbidity and mortality resulting from diarrhoea, pneumonia, and malnutrition are prevalent and national health authorities endorse breastfeeding, mothers should exclusively breastfeed their babies for 6 months, then introduce appropriate complementary foods and continue breastfeeding until at least the child’s first birthday.^10^

Elimination of VT should include efforts to retain women living with HIV in care after giving birth, and regular viral load (VL) testing and infant HIV testing during breastfeeding. According to 2024 UNAIDS global estimates, 84% [72% – > 98%] of pregnant women living with HIV were on ART to prevent HIV vertical transmission through prevention (VTP) programmes.^17^ These programmes have prevented up to 220 000 new infant infections annually. Despite these efforts, global estimates indicate that 11% of infants born to women living with HIV acquired HIV during pregnancy or the postpartum period.

Previous systematic reviews on HIV-free survival by infant feeding choice included studies conducted between 2005 and 2016, prior to the introduction of the latest WHO infant feeding and HIV treatment recommendations of lifelong ART for all.^18,19,20,21^ No recent systematic review has analysed the impact of MF on HIV transmission in HIV-exposed uninfected infants born to mothers on ART.

The aim of this systematic review is to provide a comprehensive synthesis of quantitative literature regarding infant feeding patterns, and the impact of infant feeding choices, on HIV transmission in sub-Saharan Africa in the ART (Option B+) era.

## Research methods and design

### Search methods for identification of studies

This systematic review was performed in accordance with the Preferred Reporting Items for Systematic Review and Meta-Analyses (PRISMA) guidelines,^22^ and the Patient/population, Intervention, Comparator and Outcomes (PICO) framework was used to develop a search strategy. The review considered both observational and randomised controlled trials (RCT) of mothers living with HIV on ART and their infants. The exposures were HIV infection, whether or not a mother was on ART, and feeding modality (EBF or MF). The primary outcome measure was postnatal HIV transmission.

We systematically searched the following electronic databases with no publication status restrictions: Elton B. Stephens Company (EBSCO); Academic Search Premier – Africa-Wide – Cumulative Index to Nursing and Allied Health Literature (CINAHL); Scopus; PubMed; Web of Science and Cochrane and Prospero to verify previous RCT studies; reference lists from relevant studies; and grey literature. Selection of studies was restricted to quantitative papers on infant feeding patterns published between 01 January 2013 and 31 December 2024. During this period, WHO ART and infant feeding guidelines were introduced, and guidelines updated and implemented. Two investigators (Y.R. and V.N.) conducted the search strategy to identify relevant studies. The Science Citation Index was used for searching cited references. Conference proceedings were searched in the BioSciences Information Service (BIOSIS) database (www.biosis.org), and reference lists of retrieved studies were searched for other studies. Experts in the field were contacted for unpublished or ongoing studies and trial registries, such as ClinicalTrial.gov and the WHO portal, were also searched to identify ongoing studies.

V.N. transferred and deduplicated citations from all databases in the Cadima screening tool (Julius Kühn-Institut Federal Research Centre for Cultivated Plants, Quedlinburg, Germany). The titles and abstracts were independently assessed for inclusion and conflicts in article selection were resolved through mutual discussion. V.N. and L.M. retrieved and examined full articles of potentially relevant reports for compliance with eligibility criteria.

Summary data were collected separately to assess the impact of infant feeding patterns on HIV transmission. If summary data could not be obtained, effect estimates (HIV transmission, HIV-free survival defined as the proportion of HIV exposed infants who are alive, and HIV uninfected at 18–24 months of age) were presented directly.

### Assessment of study quality and risk of bias in included studies

The review was based only on original research, reviews and conference presentations. For maintaining the quality of the review, all duplications were checked thoroughly. Only papers published in English were included. The two independent reviewers (V.N. and L.M.) assessed risk of bias and methodological quality of the included studies using the Cochrane Collaboration’s domain-based evaluation tool for assessing risk of bias and the Risk of Bias in Non-randomized Studies of Exposure (ROBINS-E) tool. The investigators reviewed all selected titles and abstracts. Assessing quality and relevance of included studies was an essential component of the review and influenced its analysis, interpretation and conclusion. Methodological components were assessed and classified as high-risk, low-risk or concerns of bias. Methodological flaws identified during risk of bias assessment were addressed in the results of included studies, where the proportion of studies assessed to be at high risk of bias was high, more caution was taken in the analysis and interpretation of the results. Any discrepancies identified were resolved through discussion. Because of the heterogeneity of the included studies, meta-analysis could not be performed.

### Ethical considerations

Ethical clearance to conduct this study was obtained from Stellenbosch University Health Research Ethics Committee (Reference number: S23/05/102 [PhD]).

## Results

**FIGURE 1:**
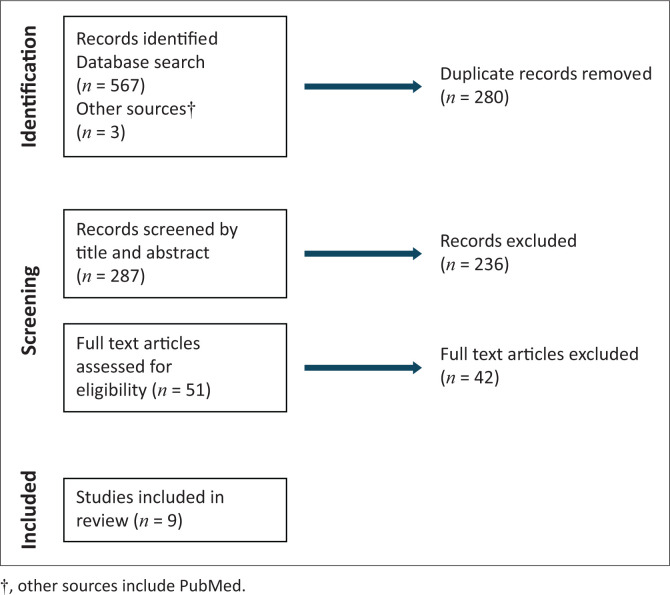
Flowchart of screening process.

The search process across several databases identified 570 studies; duplicate studies, those not evaluating HIV transmission and breastfeeding, and qualitative studies were removed (*n* = 519). V.N. and L.M. independently evaluated the full texts of 51 studies, of which nine met the inclusion criteria (Figure 1). These studies were conducted in Botswana (*n* = 1), Cameroon (*n* = 1), Ethiopia (*n* = 1), Malawi (*n* = 1), Nigeria (*n* = 2), Zambia (*n* = 1), Zimbabwe (*n* = 1), and a multicountry RCT (*n* = 1). Two were randomised clinical trials,^23,24^ three were retrospective cohorts,^14,25,26^ and four were prospective cohorts.^27,28,29,30^ The study sample sizes ranged from 170 to 2431. Timing of ART initiation (Option B+) was not uniform across studies; however, all mothers in the selected studies initiated ART before or during pregnancy and followed concurrent WHO recommendations (years 2013 to 2022). Postnatal adherence to ART data was only available in three studies.^14,27,28^ The timing of HIV infant testing was also not standardised; however, all the studies documented overall HIV transmission rate. Loss to follow-up (LTFU) was reported in seven studies, with rates ranging from 2.4% to 10.4%. In three studies,^14,24,26^ LTFU data were not presented. Exclusive breastfeeding duration was presented in seven of the nine studies, and one study only presented the median breastfeeding duration.^24^ The study settings were largely urban (8/9), with one study^24^ reporting data from both rural and urban locations.

### Infant HIV transmission

HIV VT rates were presented in all the nine studies; however, time to testing was not standardised. Three studies reported the transmission rate at 6 weeks,^14,28,30^ four at 6 months,^26,27,28,30^ and five at 12 months.^23,24,26,27,28^ Six studies reported overall HIV transmission at 18 and 24 months. Four studies did not provide confidence intervals around the calculated HIV transmission rates.^26,28,29,30^

### Postnatal HIV transmission at 6 weeks

The review findings revealed that the percentage of postnatal HIV transmission at various timepoints ranged from 0% to 2.5% in infants diagnosed at 6 weeks postpartum.^14,28,29,30^ In a secondary analysis, Volpe et al.^28^ found no breastmilk-associated HIV transmissions until 6 weeks after breastfeeding cessation. In this study from Botswana, 19 of 247 mothers had detectable VL during breastfeeding. Women living with HIV were provided government-supported ART and free formula for a year to prevent perinatal transmission for those electing exclusive formula feeding, defined as giving an infant only infant formula and no breast milk, either from birth or after a period of breastfeeding, similar to some high-income countries. Ndarukwa and Zunza^14^ reported 30 infections (*N* = 1204, HIV transmission rate 2.5% [95% CI: 1.7–3.5]), 22 (2%) of 1133 infants who were exclusively breastfeeding, and 8 (13.1%) of 61 infants who were MF, were HIV-infected. The study also showed that MF (odds ratio [OR] 3.89 [95% CI: 0.92–16.50]) and late ART initiation after delivery (OR 3.18 [95% CI: 0.42–23.94]) increased the odds of acquiring HIV, while ART adherence reduced the odds of HIV acquisition by about 99% (OR 0.01 [95% CI: 0.00–0.06]). Onubogu et.al.^30^ reported 1 of 170 infants acquiring HIV (0.7%) at 6 weeks. The mother admitted to poor ART adherence.

### Postnatal HIV transmission at 6 months

Four studies presented HIV transmission data at 6 months. Volpe et al.^28^ reported no transmission in all 19 breastfed HIV-exposed infants. Ngoma et al.^27^ also reported no transmission in 215 HIV-exposed infants. Asefa et al.^26^ reported 95% HIV-free survival in 857 infants. In the study by Onubogu et.al.,^30^ an additional infant tested positive for HIV at 6 months (*N* = 170, total transmission 1%). The mother of this infant also disclosed poor ART adherence in addition to MF.

### Postnatal transmission assessed at 12 months and overall HIV transmission rate

Four of the six studies reporting overall HIV transmission at 18 and 24 months also reported transmission at 12 months postpartum.^24,26,27,28^ Thakwalakwa et al.^23^ only reported HIV-free survival at 12 months. Noubiap, Bongoe and Demanou^25^ reported 11.6% overall transmission, with MF associated with an increased odds of HIV transmission (adjusted[a]OR 6.7 [95% CI: 1.6–28.3]; *P* = 0.009). Ngoma et al.^27^ reported two transmissions in 201 infants (1.5%) at 12 months, and an overall transmission of 4.1% (95% CI: 2.2–7.6), represented by 9 of 219 live births (including two babies who tested positive at birth). Therefore, seven transmissions occurred postpartum (postpartum transmission 3.2% [7/217]). All breastfeeding transmission that could be timed in this study occurred after 6 months of age. Volpe et al.^28^ reported no transmission; however, this result should be interpreted with caution as the sample size of 19 is too small to draw any conclusions about transmission. Assefa, Worku and Yesuf^26^ reported 93% HIV-free survival at 12 months and 84% at 18 months. Iloh et al.^29^ reported 2 (1%) HIV-positive infants out of 210 HIV-exposed infants at 18 months, lower than that reported in previous studies in Nigeria (4% – 8%). Additionally, the infants were on post-exposure antiretroviral prophylaxis with most mothers adhering to the chosen infant feeding choice.The two HIV-positive babies were both mixed fed, and their mothers had a VL ≥ 10 000 copies/mL, with significant association between VT of HIV and MF (*P* < 0.001) and maternal HIV RNA levels (*P* = 0.009).

Thakwalakwa et al.^23^ reported 90% HIV-free survival (95% CI: 87–94) at 12 months. The multicentered randomised clinical trial by Flynn et al., conducted in 14 sites in sub-Saharan Africa and India, compared the efficacy of prolonged maternal ART throughout the breastfeeding period in mother-infant dyads randomised at 6–14 days postpartum to maternal ART or infant nevirapine continued until 18 months postpartum, or breastfeeding duration, infant HIV-1 infection, or toxicity, whichever occurred first.^24^ This RCT reported a postnatal transmission rate of 0.6% (95% CI: 0.4–1.1) at 12 months, and 0.9% at 24 months (95% CI: 0.6–1.5). HIV transmission occurred in 7 of 1219 (0.57%) on maternal ART, and 7 of 1211 (0.58%) where infants received nevirapine (hazard ratio [HR] 1.0, 96% repeated CI: 0.3 – 3.1) at 12 months. The probability of an infant whose mother was on ART acquiring HIV or dying at 24 months was 2.9% (95% CI: 2.0–4.1). HIV-free survival at 24 months was 97.1%. For the infant nevirapine arm, the probability of HIV acquisition was 2.3% (95% CI: 1.5–3.4) and HIV-1-free survival was 97.7%.^24^ HIV transmission in both arms was lower than expected, and the wide 96% Reliable Change Index (RCI) (0.3–3.1) indicated very limited ability to rule out differences between the two arms because of the small numbers of infant HIV transmissions.^24^

### Adherence to maternal antiretroviral therapy

Postnatal adherence to ART was only reported in two studies.^27,28^ The WHO-recommended programme includes lifelong ART for women of childbearing age. Ngoma et al.^27^ reported an adherence level of 84.1% (190/226), with 36 of 226 women (15.9%) missing at least one dispensary visit (defined as more than 2 weeks late for a scheduled visit). In this study, adherence to ART was self-reported, and correlated poorly with attendance at dispensary visits,^27^ Volpe et al.^28^ (*N* = 19) reported a maternal ART adherence level of 87.4%, comparable to the study by Ngoma et al.^27^ Ndarukwa and Zunza^14^ reported an adherence level of 98% (*N* = 1204). Continued adherence counselling at each study visit may have enabled women to make informed decisions, improving ART adherence.

### Exclusive breastfeeding duration

Exclusive breastfeeding duration was established in seven of the nine studies (Table 1). Times for collecting data were not standardised across the seven studies, with varied reporting of EBF proportions at 6 weeks, 3, 6 or 18 months,^15,24,28,30^ and two studies reporting median EBF durations.^24,28^ Noubiap et al.^25^ reported 44.6% (50/112) EBF until 6 months postpartum, and 22.4% (25/112) MF. In the Zambian study by Ngoma et al.,^27^ 92.8% (203/219) of infants were EBF at 6 months. Similarly, in Nigeria, Onubogu et al.^30^ reported 87% (34/39) of infants being EBF at 2 weeks, declining to 21% (8/39) at 6 months in Anambra State, South East Nigeria. Early complementary feeding increased from 8% at 6 weeks to 80% at 6 months in the EBF group in Onubogu’s study.^30^ Iloh et al.^29^ reported 90.1% (64/71) EBF for 3 months. Ndarukwa et al.,^14^ in Zimbabwe, documented that 94% (1133/1204) of mothers exclusively breastfed at 6 weeks postpartum. In Malawi, Thakwalakwa et al.^23^ reported 97% (120/124) EBF for the first 6 months of life, and 89% of the children continued to be breastfed at 12 months of age. Flynn et al.^24^ (*N* = 2431) did not report EBF duration; however, they reported a median age for breastfeeding of 16 months, with 93% of infants breastfeeding at 6 months, and 86% of infants still breastfeeding by 9 months, decreasing to 34% by 18 months. Volpe et al.,^28^ in Botswana, reported a median EBF duration of 18.3 weeks (range 0.1–40.9 weeks), and a median total duration of breastfeeding of 24.7 weeks (range 0.1–86.0 weeks).

**TABLE 1:** Studies evaluating postnatal HIV transmission in infants born to HIV-positive mothers and the impact of infant feeding choice on postnatal HIV transmission in the ART (Option B+) era.

Study authors	Year	Country	Study Site	Study design	Sample size†	Postnatal adherence to ART	EBF duration	HIV transmisssion rate at 6 weeks/ HFS	HIV transmisssion rate/ HFS at 6 months	HIV transmisssion rate/ HFS at 12 months	Overall HIV transmisssion rate/HFS at 18 months/ 24 months	% LTFU
Noubiap et al.	2013	Cameroon	Urban	Retrospective cohort	125	‡	50.6% until 6 months	‡	‡	‡	11.6%	10.4%
Ngoma et al.	2015	Zambia	Urban	Prospective cohort	279	84.1%	92.8% EBF until 6 months	‡	0%	1.5%	4.1%	13.0%
Volpe et al.	2022	Botswana	Urban	Prospective cohort	19§	87.4%	Median duration EBF 18.3 weeks (range 0.1 – 40.9 weeks)	0.0%	0%	0.0%	0.0%	0.0%
Iloh et al.	2015	Nigeria	Urban	Prospective cohort	210	‡	90.1% at 3 months	‡	‡	‡	1.0%	0.0%
Ndarukwa & Zunza	2019	Zimbabwe	Urban	Retrospective cohort	1204	98.0%	94.0% at 6 weeks	2.5%	‡	‡	‡	‡
Assefa et al.	2017	Ethiopia	Urban	Retrospective cohort	857	‡	‡	‡	HFS 95%	HFS 93%	HFS 84%	‡
Thakwalakwa et al.	2014	Malawi	Urban	Randomised clinical trial	248	‡	97.0% EBF for the first 6 months	‡	‡	HFS 90%	‡	2.4%
Onubogu et al.	2015	Nigeria	Urban	Prospective cohort study	170	‡	87.0% at 2 weeks 21.0% at 6 months	0.7%	1%	‡	‡	4.0%
Flynn et al.	2018	Malawi, South Africa, Tanzania, Uganda, Zambia, Zimbabwe, and India	Both rural and urban	Randomised clinical trial	2431	‡	EBF ‡	‡	‡	0.6% postnatal infection rate at 12 months	0.9% infection rate at 24 months	‡

Note: Please see the full reference list of the article, Ndarukwa V, Cotton M, Zunza M, Mupfumi L, Amukugo H. Postnatal HIV vertical transmission and the impact of infant feeding choice in the ART era. S Afr J HIV Med. 2025;26(1), a1738. https://doi.org/10.4102/sajhivmed.v26i1.1738, for more information.

HEU, HIV-1 exposed uninfected; HFS, HIV free survival; LFTU, Loss to follow-up; EBF, exclusive breastfeeding; MF, mixed feeding; EBMS, exclusive breast milk substitute; PCR, polymerase chain reaction; ART, antiretroviral therapy.

† , HIV+ women and HEU;

‡ , data not presented;

§ , subset of larger cohort *N* = 247.

### Grade profile

The assessment of the studies included in this analysis was based on study limitations and risk of bias including reporting, selection, detection and attrition bias using the ROBINS-E tool. The two RCTs were rated high quality, and the remainder were rated low quality, as a result of being observational. Some were further downgraded to very low quality because of a high risk of selection and reporting bias. Heterogeneity was also observed across studies which did not assess HIV transmission rates at the same time points. Therefore, pooled analysis could not be undertaken, leading to further downgrading of the studies.

## Discussion

Current WHO guidelines recommend lifelong ART for pregnant and breastfeeding women living with HIV. Exclusive breastfeeding is recommended in the first 6 months of life, adding complementary feeding until 24 months of age or beyond. The decision to continue breastfeeding is based on comparing the risk of infants acquiring HIV through breastfeeding, with the increased risk of death from malnutrition, diarrhoea and pneumonia if not exclusively breastfed. In the included studies, the mothers were encouraged to exclusively breastfeed up to 6 months of age, following the WHO 2010 recommendations.^10^ This systematic review reveals a low risk of postnatal transmission of HIV in the presence of ART and EBF. Transmission was estimated at between 0% and 2.5% by age 6 months, indicating ART efficacy when recommended WHO feeding and treatment guidelines are implemented. However, MF infants were found to be at a higher risk of seroconverting compared to EBF infants, even in the presence of ART.^14,30^

The overall HIV transmission was measured at varying time points across the studies, at 12, 18 and 24 months. Of the studies that provided transmission rates at 12 months, the estimates ranged between 0% and 7% (*N* = 3815). Higher HIV transmission rates were obtained at 18 to 24 months, ranging between 0% and 11.6% (*N* = 3824). The high HIV transmission rate of 11.6% reported in the Cameroon study^25^ was associated with MF, missed antenatal visits, and poor maternal compliance to VTP programmes, as well as suboptimal early infant diagnosis implementation. Several mothers and infants were referred for HIV diagnosis after presenting with clinical manifestations of HIV.

ART adherence is critical for achieving infant HIV elimination. All studies in this review provided lifelong ART and recommended EBF up to 6 months, adding complementary feeding until 24 months and beyond; however, this did not translate to all mothers adhering to treatment for the entire period. Only three studies reported data on maternal adherence. Ngoma et al.^27^ reported 84.1% maternal adherence, while even higher ART adherence levels were reported by Volpe et al.^28^ (87.4%) and Ndarukwa and Zunza^14^ (98%). The higher ART adherence levels in the Botswana study could be attributed to the frequent monitoring of VL, coupled with counselling on ART adherence and safe infant feeding. In contrast, the adherence to ART in the study by Ndarukwa and Zunza^14^ was retrieved from existing clinical records, and reporting bias could have been introduced; therefore, these results should be interpreted with caution. Volpe et al.^28^ reported no HIV transmission in their small cohort (*N* = 9), while Ndarukwa and Zunza^14^ reported a 2.5% HIV transmission at 6 weeks postpartum. The primary outcome in Ngoma et al.’s study^27^ was HIV-free survival after extended breastfeeding (12 months), followed by gradual cessation of breastfeeding. The participants self-reported adherence to breastfeeding and ART, which was unreliable and correlated poorly with attendance at dispensary visits. An estimated 16% of the mothers missed at least one dispensary visit. The study reported that 4 of 35 (11.4%) women who missed a visit compared to 5 of 184 (2.7%) women who did not miss a visit transmitted HIV, highlighting the importance of therapy adherence in averting HIV postnatal transmission. Compliance/adherence bias was introduced as adherence to ART data was not provided in most studies. Where self-reported adherence rates were reported as in the Zambia study, caution should be applied when interpreting study results.

There were limited data on the levels of viral suppression amongst these mothers on ART.

Eight of the nine studies documented MF before the infant reached 6 months postpartum, consistent with literature from studies conducted in Africa. Exclusive breastfeeding was highest among women who were receiving counselling and support as in Botswana and lowest among those with limited healthcare support and counselling, as in Cameroon. Noubiap et al.^25^ also found that only 50.6% of infants were exclusively breastfed at 6 months, while 22.4% of infants received MF. In Noubiap’s study, as in similar studies conducted in African settings, mothers who decided to exclusively formula feed their infants faced stigma, which likely forced them to mixed feed their babies. Infant feeding practices were self-reported, leading to possible reporting bias.

Several studies have shown a four-fold increase in risk HIV transmission associated with MF in the presence of ART. In agreement with existing literature, two of these studies reporting HIV transmission in the context of MF also showed higher transmission rates^14,25^ than EBF and exclusive breast milk substitute. The Cameroon study by Noubiap et al.^25^ showed that HIV VT was significantly associated with MF (aOR 6.7, 95% CI: 1.6–28.3; *P* = 0.009). Ndarukwa and Zunza,^14^ in Zimbabwe, revealed an almost four-fold increase in risk of HIV transmission in MF infants compared to EBF infants at 6 weeks postpartum (OR 3.89), aligning with findings in earlier studies.^15,16^ These study findings support the recommendation that breastfeeding mothers should receive counselling and support on optimal infant feeding practices, including EBF for the first 6 months of life.

Overall, timely and effective communication and counselling regarding maternal ART adherence, infant feeding practices, frequent VL monitoring, frequent infant HIV testing, and offering advice to switch to exclusive formula feeding in the presence of detectable VL, enabled women to make informed decisions about infant feeding and potentially improve ART adherence and lower HIV VT. In contrast, suboptimal ART adherence increased the infant’s exposure to HIV in breastmilk and thus increased VT.

## Conclusion

The findings of the systematic review showed that mothers on lifelong ART and EBF, as per the prevailing WHO recommendations, have a lower risk of postnatal HIV transmission; however, in the presence of MF, this risk is increased. The importance of integrating public health programmes to support women living with HIV to adhere to treatment and EBF recommendations was also revealed.

The review had several limitations which lowered the quality of evidence. These included considerable heterogeneity in study methodologies, such as varying timing of ART initiation and infant HIV testing. Standardised protocols for HIV diagnosis and treatment must be implemented for VT rates to be accurately determined in sub-Saharan Africa and worldwide. Testing for paediatric HIV should ideally be conducted at birth, 6–10 weeks postpartum, 6 months postpartum, and 6 weeks after breastfeeding cessation.

Because of limited data, gaps still exist in establishing the actual risk of postnatal HIV transmission associated with MF in mothers who are adhering to ART and have suppressed VLs in different settings (urban and rural). Prospective cohorts with larger sample sizes are required to evaluate the impact and efficacy of Option B+ and EBF in VTP programmes in Africa, including impact of feeding options on infant growth and morbidity.
